# LncRNA MAYA promotes iron overload and hepatocyte senescence through inhibition of YAP in non‐alcoholic fatty liver disease

**DOI:** 10.1111/jcmm.16764

**Published:** 2021-06-30

**Authors:** Ping Yuan, Xiaoyu Qi, Anping Song, Mingyue Ma, Xinbei Zhang, Chunfeng Lu, Mianli Bian, Naqi Lian, Jianling He, Shuguo Zheng, Huanhuan Jin

**Affiliations:** ^1^ Department of Pharmacology School of Pharmacy Wannan Medical College Wuhu China; ^2^ School of Pharmacy Nantong University Nantong China; ^3^ Nanjing University of Chinese Medicine Nanjing China; ^4^ Ministry of Natural Resources Third Institute of Oceanography Xiamen China

**Keywords:** cellular senescence, iron overload, MAYA, non‐alcoholic fatty liver disease, Yes‐associated protein

## Abstract

Although recent evidence has shown that hepatocyte senescence plays a crucial role in the pathogenesis and development of non‐alcoholic fatty liver disease (NAFLD), the mechanism is still not clear. The purpose of this study was to investigate the signal transduction pathways involved in the senescence of hepatocyte, in order to provide a potential strategy for blocking the process of NAFLD. The results confirmed that hepatocyte senescence occurred in HFD‐fed Golden hamsters and PA‐treated LO2 cells as manifested by increased levels of senescence marker SA‐β‐gal, p16 and p21, heterochromatin marker H3K9me3, DNA damage marker γ‐H2AX and decreased activity of telomerase. Further studies demonstrated that iron overload could promote the senescence of hepatocyte, whereas the overexpression of Yes‐associated protein (YAP) could blunt iron overload and alleviate the senescence of hepatocyte. Of importance, depression of lncRNA MAYA (MAYA) reduced iron overload and cellular senescence via promotion of YAP in PA‐treated hepatocytes. These effects were further supported by in vivo experiments. In conclusion, these data suggested that inhibition of MAYA could up‐regulate YAP, which might repress hepatocyte senescence through modulating iron overload. In addition, these findings provided a promising option for heading off the development of NAFLD by abrogating hepatocyte senescence.

## 1 INTRODUCTION

Non‐alcoholic fatty liver disease (NAFLD) is the most frequent chronic liver disease in many areas of the world. According to the data published recently, the overall prevalence of NAFLD was 29.8% in China.^1^ NAFLD, a multi‐system disease related to metabolic disorders, is associated with liver injury^2^ and may progress to advanced liver fibrosis, cirrhosis and even hepatocellular carcinoma if not controlled effectively. Up until now, there is no efficacious drugs for NAFLD, due to the fact that the molecular mechanism of NAFLD has not been elucidated completely. Therefore, it is essential for the discovery of effective target drugs to explore the novel therapeutic targets of NAFLD.

The senescence of hepatocyte plays an important role in the pathogenesis of NAFLD, and thus, hepatocyte senescence‐related molecules have been becoming therapeutic targets for the treatment of NAFLD.^3^ Cellular senescence, a consequence of replicative senescence,^4^, ^5^ is typically characterized by telomere dysfunction and shortening, up‐regulation of ageing‐related genes, accumulation of DNA damage and limits the proliferative potential of damaged cells through the induction of cell cycle arrest.^6^, ^7^ It was reported that hepatocyte senescence could promote hepatic steatosis in non‐alcoholic fatty liver disease and elimination of ageing hepatocytes or inhibition of hepatocyte senescence might be effective strategies for the amelioration of NAFLD.^8^ In recent years, a host of studies have shown that the pathogenesis of non‐alcoholic fatty liver disease is frequently associated with iron overload.^9^, ^10^, ^11^ As an organ for iron recycling, storage and iron‐containing enzyme synthesis, liver plays a key role in the process of iron metabolism. The disorder of iron metabolism leads to excessive iron deposition in the liver of patients with NAFLD, which is usually called iron overload and thought to be involved in oxidative stress and mitochondrial dysfunction, two pathological factors associated with cellular senescence.^12^, ^13^, ^14^, ^15^ However, there is no evidence that iron overload can induce hepatocyte senescence in NAFLD.

At present, little is known about the specific signal pathways through which pathological iron overload promotes liver cell senescence. Yes‐associated protein (YAP), a major downstream effector of the Hippo signalling pathway, has been reported to be involved in regulating cellular senescence.^16^, ^17^, ^18^ However, the relationship between YAP and iron overload has not been explored. A recent study has reported that long non‐coding RNA (lncRNA) *MAYA* (*MST1/2‐Antagonizing for YAP Activation*) is involved in YAP activation.^19^ LncRNAs, a novel category of non‐coding RNAs, exert its regulatory functions through specific interactions with proteins, including transcriptional factors/coactivators, epigenetic modifiers and RNP complexes.^20^ A few of top international academic journals showed that the regulation of lncRNA plays a considerable role in occurrence and progression of NAFLD.^21^, ^22^ In the early stage of this study, we verified that palmitic acid (PA) induced cellular senescence in LO2 cells. In addition, we found that the expression of MAYA increased in PA‐treated LO2 cells. In the present study, we performed both in vivo and in vitro experiments to investigate the signal transduction pathways involved in hepatocyte senescence from the aspect of MAYA/YAP regulation of iron overload.

## 2 MATERIALS AND METHODS

### 2.1 | **Reagents and antibodies**

These reagents were applied in this study: Oil Red O (Sigma, St. Louis, MO, USA); Senescence‐associated β‐galactosidase (SA‐β‐gal) staining kit (Cell Signaling Technology, Danvers, MA, USA); Lipofectamine2000 reagent, TRIzol reagent (Invitrogen, Thermo Fisher Scientific, Waltham, MA, USA); RevertAid First Strand cDNA Synthesis Kit (Thermo Scientific™, Thermo Fisher Scientific, Waltham, MA, USA); PowerUp™ SYBR™ Green Master Mix (Applied Biosystems™, Thermo Fisher Scientific, Waltham, MA, USA); Iron Colorimetric Assay Kit (APPLYGEN, Beijing, China); PA, Ferric ammonium citrate (FAC) (Solarbio, Beijing, China); YAP CRISPR Activation Plasmid (Santa Cruz Biotechnology, Santa Cruz, CA, USA); Fetal bovine serum (ExCell Bio, Shanghai, China); Dulbecco's modified Eagle's medium (DMEM) (GIBCO BRL, NY, USA). The primary antibodies were used in this study as follows: p16, p21, histone H3 trimethylated at lysine 9 (H3K9me3), YAP, phosphorylation of histone H2AX (γ‐H2AX) (Cell Signaling Technology, Danvers, MA, USA); Telomeric repeat binding factors 2 (TRF2) (Santa Cruz Biotechnology, Santa Cruz, CA, USA); Telomerase reverse transcriptase (TERT), Telomeric repeat binding factors 1 (TRF1) (Bioss, Beijing, China); β‐actin (ProteinTech Group, Chicago, IL, USA).

### 2.2 | **Experimental animal procedures**

All experimental protocols involving animal acquired the approval of the Animal Care and Use Committee of Wannan Medical College (Wuhu, China), and all animals received humane care according to the National Institutes of Health (USA) guidelines. LVG Golden Syrian hamsters (80 ~ 110 g bodyweight) were purchased from Beijing Vital River Laboratory Animal Technology Co., Ltd. A total of 16 hamsters were adaptively fed for a week and then randomly divided into 2 groups (n = 8) as follows: Group 1 was the negative control (NC) in which hamsters were given a standard diet and hamsters in Group 2 were given a high‐fat diet for 8 weeks. At the end of experiments, hamsters were killed after being anaesthetised by intraperitoneal injection with pentobarbital (50 mg/kg). A small portion of the liver was fixed in a 10% neutral buffered formalin for histological analysis, and the others were stored at −80℃ for further analyses, such as Oil Red O, SA‐β‐gal. Eight‐week‐old male C57BL/6J mice (18 ~ 20 g bodyweight, Nanjing Qinglongshan Experimental Animal Company) were randomly divided into 5 groups (n = 8) and given a standard diet in group1 and group 2 or a high‐fat diet in the other groups after adaptive feeding for 1 week. After 10 weeks, mice in groups 2 and 3 were injected with sh‐NC, and groups 4 and 5 were injected with sh‐MAYA‐1 and sh‐MAYA‐2 through the tail veil, respectively. The lentiviral3‐GFP‐shRNA specifically targeting MAYA was designed and synthesized by GenePharma (Shanghai, China), and nonspecific shRNA was used as negative control (sh‐NC). Lentiviral vector (1 × 10^9^ TU/ml) was injected into mice for 24 days (once per 8 days). At the end of experiments, all the animals were killed after being anaesthetised by intraperitoneal injection with pentobarbital. Part of the liver was fixed in a 10% neutral buffered formalin for histological analysis and the others were stored at −80℃ for further analysis. The sequences of MAYA shRNA (mice) were as following: sh‐MAYA#A: 5'‐GCACATAGCTCTTGTCTTTAG‐3'; sh‐MAYA#B: 5'‐GCCCAATTAAGGCAAGTAAGG‐3'.

### 2.3 | **Cell culture**

The human immortalized normal hepatocyte cell line LO2 cells (Cell Bank of Chinese Academy of Sciences, Shanghai, China) were cultured in Dulbecco's modified Eagle's medium (DMEM; Invitrogen, Grand Island, NY, USA) with 10% foetal bovine serum and 1% penicillin and streptomycin, and grown in the 5% CO_2_ incubator at 37℃.

### 2.4 | **Haematoxylin and eosin (H&E) staining and immunohistochemistry staining**

Liver tissues fixed in 10% neutral buffered formalin were embedded in paraffin and cut into 4 μm thick slices. H&E staining and immunohistochemistry staining were performed as described in our previous study.^23^

### 2.5 | **Oil Red O staining**

Frozen section of liver tissues and cultured LO2 cells in 24‐well plates were subjected to Oil Red O staining as previously described.^13^

### 2.6 | **Iron content assay**

The iron content of liver tissue was measured by Tissue Iron Content Colorimetric Assay Kit (Nanjing Jiancheng Bioengineering Institute, Nanjing, China) following the manufacturer's instructions. Cellular iron content was measured by Iron Content Assay Kit (APPLYGEN, Beijing, China). LO2 cells in a 24‐well plate were lysed on ice with cell lysate for 2 hours, mixed with 4.5% potassium permanganate solution at 60℃ for 1 hour and added with iron detection reagent, and the absorbance is measured at 550 nm. All these experiments were performed in triplicate.

### 2.7 | **Cell transfection with YAP plasmid or sh‐MAYA**

YAP activation plasmid (1 μg) and Lipofectamine2000 reagent (5 μl) were mixed with 200 μl DMEM (without serum and antibiotics) for 5 min at room temperature, respectively. The above two solutions were mixed fully at room temperature for 15 min. Following incubation with the transfection complex for 6 h at 37℃, LO2 cells were re‐incubated with complete medium for 24 h and collected for further experiment. Empty vector plasmid was designed as a negative control. The transfections of sh‐NC, sh‐MAYA#1 and sh‐MAYA#2 were performed in accordance with the protocol of YAP activation plasmid transfection. The sequences of MAYA shRNA (human) were as following: sh‐MAYA#1:5'‐TGCTGTTGACAGTGAGCGAGTGGGTGAAACACACAGGGAATAGTGAAGCCACAGATGTATTCCCTGTGTGTTTCACCCACCTGCCTACTGCCTCGGA‐3'; sh‐MAYA#2:5'‐TGCTGTTGACAGTGAGCGAGGGAACAACGCAGACAACATATAGTGAAGCCACAGATGTATATGTTGTGCGCGTTGTTCCCGTGCCTACTGCCTCGGA‐3'.

### 2.8 | **Quantitative Real‐time polymerase chain reaction (qRT‐PCR)**

Total RNA was extracted with TRIzol reagent, and cDNA was obtained using the RevertAid First Strand cDNA Synthesis Kit according to the protocol of manufacturer. Conditions for the quantitative polymerase chain reaction were as follows: hold stage was 50℃ for 2 min and 95℃ for 2 min; cycling was 40 cycles of 95℃ for 15 sec, 60℃ for 15 sec and 72℃ for 1 min; and melting curve record was 95℃ for 15 sec, 60℃ for 1min and 95℃ for 15 sec. The mRNA levels of correlative factors were determined using the 2^−delta delta CT^ analysis method. Glyceraldehyde phosphate dehydrogenase (GAPDH) was applied as the endogenous control. Results were from triplicate experiments. The following primer sequences of examined genes in our experiments were used:

TERT (hamster):

(forward) 5’‐AGGTCAAGAATGCAGGAATGACA‐3’,

(reverse) 5’‐AGTGGTGAGGCTACAATGCC‐3’;

TRF1 (hamster):

(forward) 5’‐GATAGGCCAGATGCCACCAA‐3’,

(reverse) 5’‐AAGGCGTTCTTGTGAGACCTTA‐3’;

TRF2 (hamster):

(forward) 5’‐ ACCCTCTCCTCCTCCCACTA‐3’,

(reverse) 5’‐TCTTCACCTGGTGCCTGAAC‐3’;

p16 (hamster):

(forward) 5’‐ TGGTCACTGTGAGGATTCAGC‐3’,

(reverse) 5’‐ TGCCCATCATCATCACCTGGTC −3’;

p21 (hamster):

(forward) 5’‐ CAGAATAAAAGGTGCCACAGG‐3’,

(reverse) 5’‐ AATCTTCAGGCCGCTCAGAC‐3’;

GAPDH (hamster):

(forward) 5’‐ GACATCAAGAAGGTGGTGAAGCA‐3’,

(reverse) 5’‐CATCAAAGGTGGAAGAGTGGGA‐3’;

TERT (human):

(forward) 5’‐CGGAAGAGTGTCTGGAGCAA‐3’,

(reverse) 5’‐ GGATGAAGCGGAGTCTGGA‐3’;

TRF1 (human):

(forward) 5’‐GACACTGGGGAGGTAGGGT‐3’,

(reverse) 5’‐GCTAACAAACCTGCCCATG‐3’;

TRF2 (human):

(forward) 5’‐ TCAATCGCTGGGTGCTCAA‐3’,

(reverse) 5’‐ TCAATCGCTGGGTGCTCAA‐3’;

p16 (human):

(forward) 5’‐ GGAGTTAATAGCACCTCCTCC‐3’,

(reverse) 5’‐ TTCAATCGGGGATGTCTGAGG‐3’;

p21 (human):

(forward) 5’‐ GTCAGTTCCTTGTGGAGCCG‐3’,

(reverse) 5’‐ GAAGGTAGAGCTTGGGCAGG‐3’;

GAPDH (human):

(forward) 5’‐ CTTCTTTTGCGTCGCCAGCCGA‐3’,

(reverse) 5’‐ ACCAGGCGCCCAATACGACCAA‐3’;

MAYA (human):

(forward) 5’‐ GCAGCAGAACTACCCTCCAG‐3’,

(reverse) 5’‐ ACCCTCTCCTCCTCCCACTA‐3’;

MAYA (mice):

(forward) 5’‐ CCCATGGGCCTTTTCCAGAT‐3’,

(reverse) 5’‐ ACGACTCCCAGTCTCTCTCC‐3’;

GAPDH (mice):

(forward) 5’‐ AGGTCGGTGTGAACGGATTTG‐3’,

(reverse) 5’‐ TGTAGACCATGTAGTTGAGGTCA‐3’.

### 2.9 | **Immunofluorescence staining**

Immunofluorescence staining of liver tissues or LO2 cells was performed in accordance with the standard procedure of previous report.^24^ DAPI (Beyotime, Shanghai, China) stain solution was used to locate the nucleus of hepatocytes in vivo and in vitro.

### 2.10 | SA‐β‐gal staining

SA‐β‐gal staining was performed based on the manufacturer's protocol. In brief, LO2 cells cultured in 24‐well plates were washed with phosphate buffered saline (PBS) and fixed with 0.5ml fixative solution. The fixed cells were rinsed 2 times with PBS and incubated with 0.5ml/well of the β‐galactosidase staining solution at 37℃ overnight in a dry incubator (no CO_2_). Frozen sections of liver tissues were performed in accordance with that for LO2 cells.

### 2.11 | Western blot analysis

LO2 cells or liver tissues were lysed in RIPA buffer containing protease inhibitors, and the protein concentration was quantified by using a BCA protein assay kit (Beyotime, Shanghai, China). The experimental procedure of Western blot was as described in our previous research.^23^ Representative blots were from three independent experiments.

### 2.12 | Statistical analysis

Results were presented as mean ±SEM, and statistical analysis was performed using GraphPad Prism 5.0 (GraphPad Software, San Diego, CA, USA). The significance of difference was determined by one‐way analysis of variance with the post hoc Dunnett's test. Values of *P* <.05 were considered statistically significant.

## 3 RESULTS

### 3.1 | **The senescence of hepatocyte occurred in the liver of hamsters with non‐alcoholic fatty liver disease**

**FIGURE 1:**
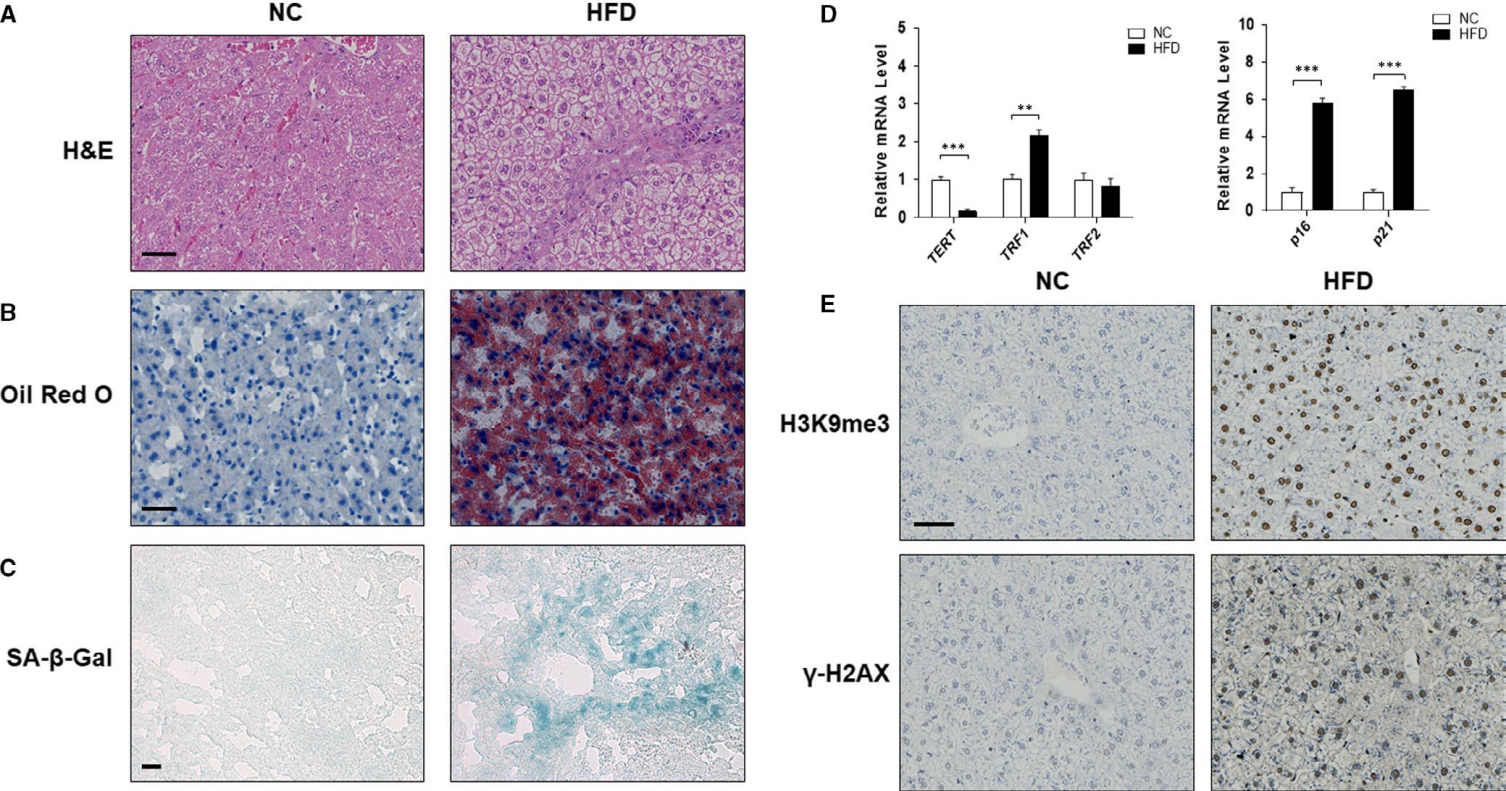
The senescence of hepatocyte occurred in the liver of hamsters with non‐alcoholic fatty liver disease. Male LVG Golden Syrian hamsters were randomly separated into following two groups: group 1, NC group (Standard diet); group 2, HFD group. Liver sections were stained with H&E (A), Oil Red O (B) and SA‐β‐gal (C). Representative photographs are shown. Scale bar, 50 μm (A,B), 100 μm (C). (D) qRT‐PCR analyses of mRNA expression of TERT, TRF1, TRF2, p16 and p21 respectively. Data are represented as mean ±SEM. Significance: ^∗∗^ *P* <.01 versus group 1, ^∗∗∗^ *P* <.001 versus group 1. (E) Protein expression of H3K9me3 and γ‐H2AX in vivo was investigated by immunohistochemistry on paraffin sections of hamster livers. Scale bar, 50 μm

Golden hamster is an ideal model for NAFLD because the distribution and metabolism of blood lipids are similar to human. In order to clarify the hepatocyte senescence in NAFLD liver, we used LVG Golden Syrian hamsters to establish an in vivo model of NAFLD with a high‐fat diet (HFD).^25^ Photomicrographs of H&E staining showed that HFD feeding induced ballooning degeneration in liver tissues, accompanied by conspicuous cell swelling, cytoplasmic vacuolation and inflammatory infiltration (Figure 1A). More accumulation of hepatic lipid droplets was observed in NAFLD hamsters than in control group as assessed by Oil Red O staining (Figure 1B). SA‐β‐gal activity assay was used to detect cellular senescence and the results showed that SA‐β‐gal‐positive hepatocytes increased significantly in the model group (Figure 1C).

It is well known that telomere shortening and telomerase inhibition lead to replicative senescence.^26^ Telomeric Repeat Binding Factor 1 and 2 (TRF1 and TRF2) are responsible for the major telomerase activity.^27^, ^28^ TRF1 negatively regulates telomere length, whereas TRF2 maintains telomere structure.^29^, ^30^ Therefore, we detected the mRNA levels of TERT, TRF1 and TRF2 by qRT‐PCR and found that HFD feeding induced a significant down‐regulation of TERT mRNA and up‐regulation of TRF1 mRNA, whereas the level of TRF2 mRNA remained unchanged (Figure 1D). Consistently, the analyses of senescence‐associated genes showed that HFD treatment up‐regulated the expression of senescence markers p16 and p21 (Figure 1D). In addition, the protein levels of heterochromatin marker H3K9me3 and DNA damage marker γ‐H2AX were increased in HFD feeding hamsters (Figure 1E), providing further evidence that the cells enter into senescence.^31^ Taken together, these results strongly illustrated that HFD‐induced lipid accumulation may elicit hepatocyte senescence in vivo.

### 3.2 | PA promoted cellular senescence in human hepatocyte LO2 cells

**FIGURE 2:**
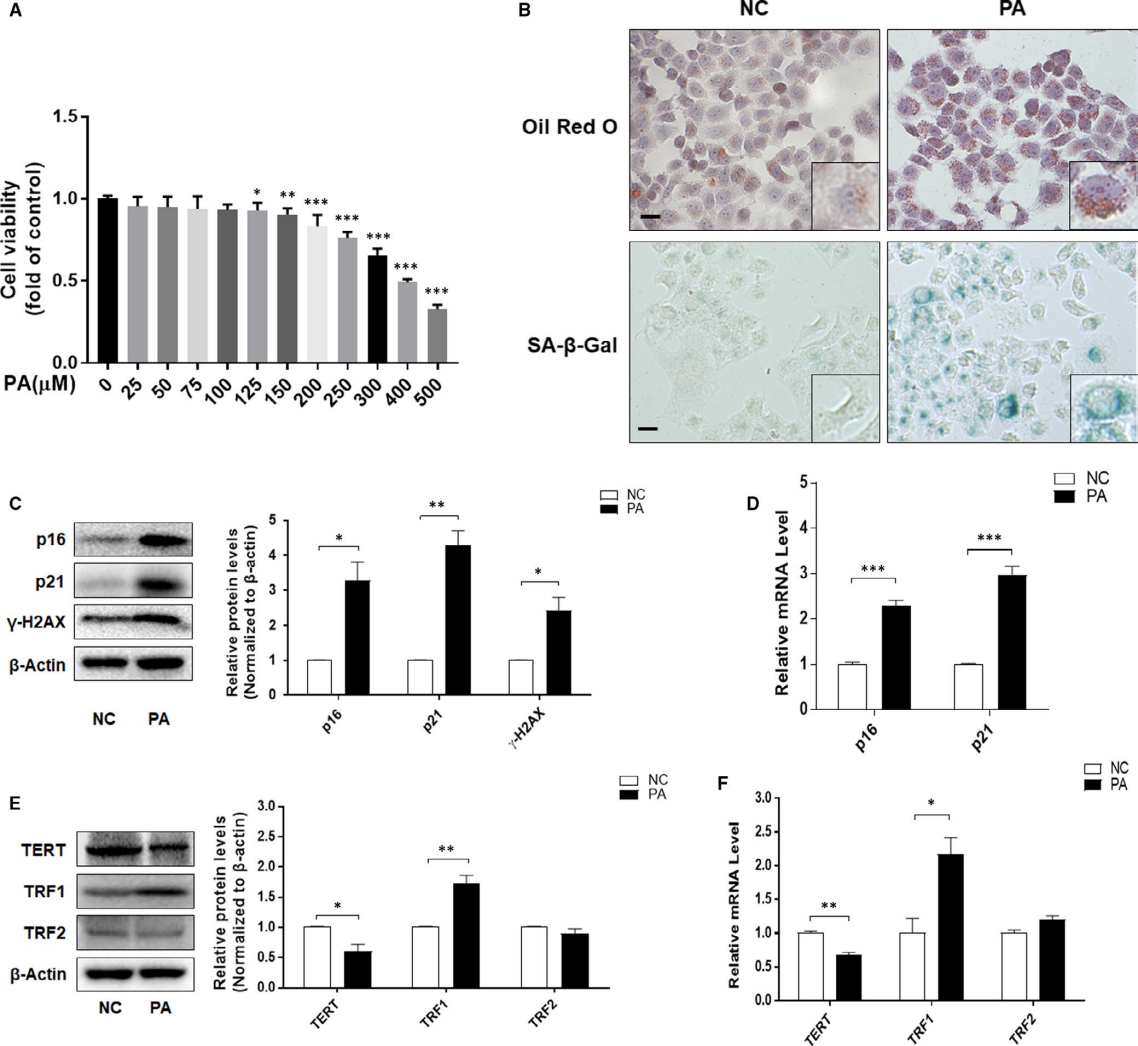
PA promoted cellular senescence of human hepatocyte LO2 cells. A, Cell Count Kit‐8 analysis of the cell viability of LO2 cells. Significance: ^∗^ *P* <.05 versus NC, ^∗∗^ *P* <.01 versus NC, ^∗∗∗^ *P* <.001 versus NC. B, Representative image of Oil Red O and SA‐β‐gal staining of LO2 cells under the indicated conditions. Scale bar, 100 μm. C, E, Western blot analyses of p16, p21, γ‐H2AX, TERT, TRF1 and TRF2 in LO2 cells. Data are represented as mean ±SEM. Significance: ^∗^ *P <*.05 versus NC, ^∗∗^ *P <*.01 versus NC. D, F, qRT‐PCR analyses of p16, p21, TERT, TRF1 and TRF2 in LO2 cells. Data are represented as mean ±SEM. Significance: ^∗^ *P* <.05 versus NC, ^∗∗^ *P* <.01 versus NC, ^∗∗∗^ *P <*.001 versus NC

Human immortalized hepatocyte LO2 cells were incubated with 0.15 mmol/L PA for 24 h to establish a model of NAFLD in vitro (Figure 2A). The results of Oil Red O and SA‐β‐gal staining showed that PA treatment induced lipid accumulation and cellular senescence in LO2 cells (Figure 2B), with both mRNA and protein levels of senescence markers p16 and p21 up‐regulated markedly (Figure 2C, D). The protein level of DNA damage marker γ‐H2AX was increased by PA (Figure 2C). In addition, PA treatment caused a significant down‐regulation of TERT expression and up‐regulation of TRF1 expression in LO2 cells, whereas the expression of TRF2 remained unchanged (Figure 2E, F). These findings were in accordance with in vivo results, indicating that extensive fat accumulation could cause hepatocyte senescence.

### 3.3 | Iron overload mediated PA‐induced cellular senescence in LO2 cells

**FIGURE 3:**
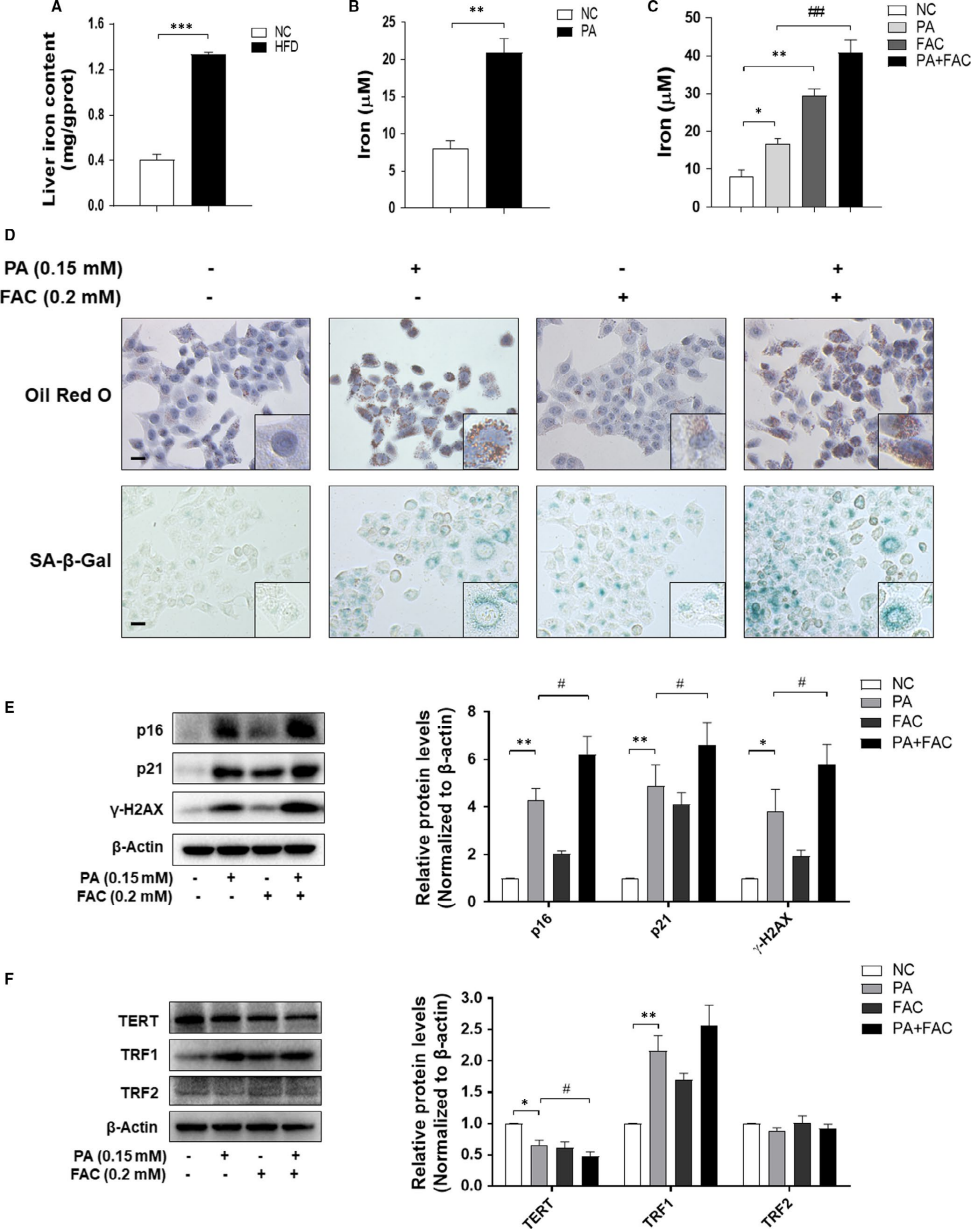
Iron overload mediated PA‐induced cellular senescence in LO2 cells. A, Determination of iron content by Tissue Iron Content Colorimetric Assay Kit. Significance: ^∗∗∗^ *P <*.001 versus NC. B, C, The iron content of LO2 cells was evaluated by Iron Content Assay Kit. Significance: ^∗^ *P <*.05 versus NC, ^∗∗^ *P <*.01 versus NC, ^##^ *P <*.01 versus PA treatment. D, Representative image of Oil Red O and SA‐β‐gal staining of LO2 cells under the indicated conditions. Scale bar, 100 μm. E, F, The protein levels of p16, p21, γ‐H2AX, TERT, TRF1 and TRF2 were determined by Western blot. Data are represented as mean ±SEM. Significance: ^∗^ *P <*.05 versus NC, ^∗∗^ *P <*.01 versus NC, ^#^ *P <*.05 versus PA treatment

To elucidate the correlation between NAFLD and iron overload, we first detected whether there is iron overload in in vivo and in vitro NAFLD models. The results showed that the iron contents in livers of NAFLD hamsters and PA‐treated LO2 cells were significantly higher than those in the control groups (Figure 3A, B). To further evaluate the effect of iron overload on the accumulation of lipid droplets and cellular senescence, we treated LO2 cells with FAC (0.2 mmol/L) for 24 hours to construct iron overloaded‐model^13^ (Figure 3C). Oil red O and SA‐β‐gal staining displayed that FAC increased intracellular lipids deposition and cellular senescence in PA‐treated LO2 cells (Figure 3D), with the up‐regulated protein levels of p16, p21 and γ‐H2AX when compared with PA‐treated group (Figure 3E). Consistently, exposure to FAC further decreased the protein level of TERT and increased the protein level of TRF1 in PA‐treated LO2 cells (Figure 3F). These results indicated that iron overload mediated PA‐induced cellular senescence in LO2 cells.

### 3.4 | **Overexpression of YAP inhibited iron overload and cellular senescence in PA‐treated LO2 cells**

**FIGURE 4:**
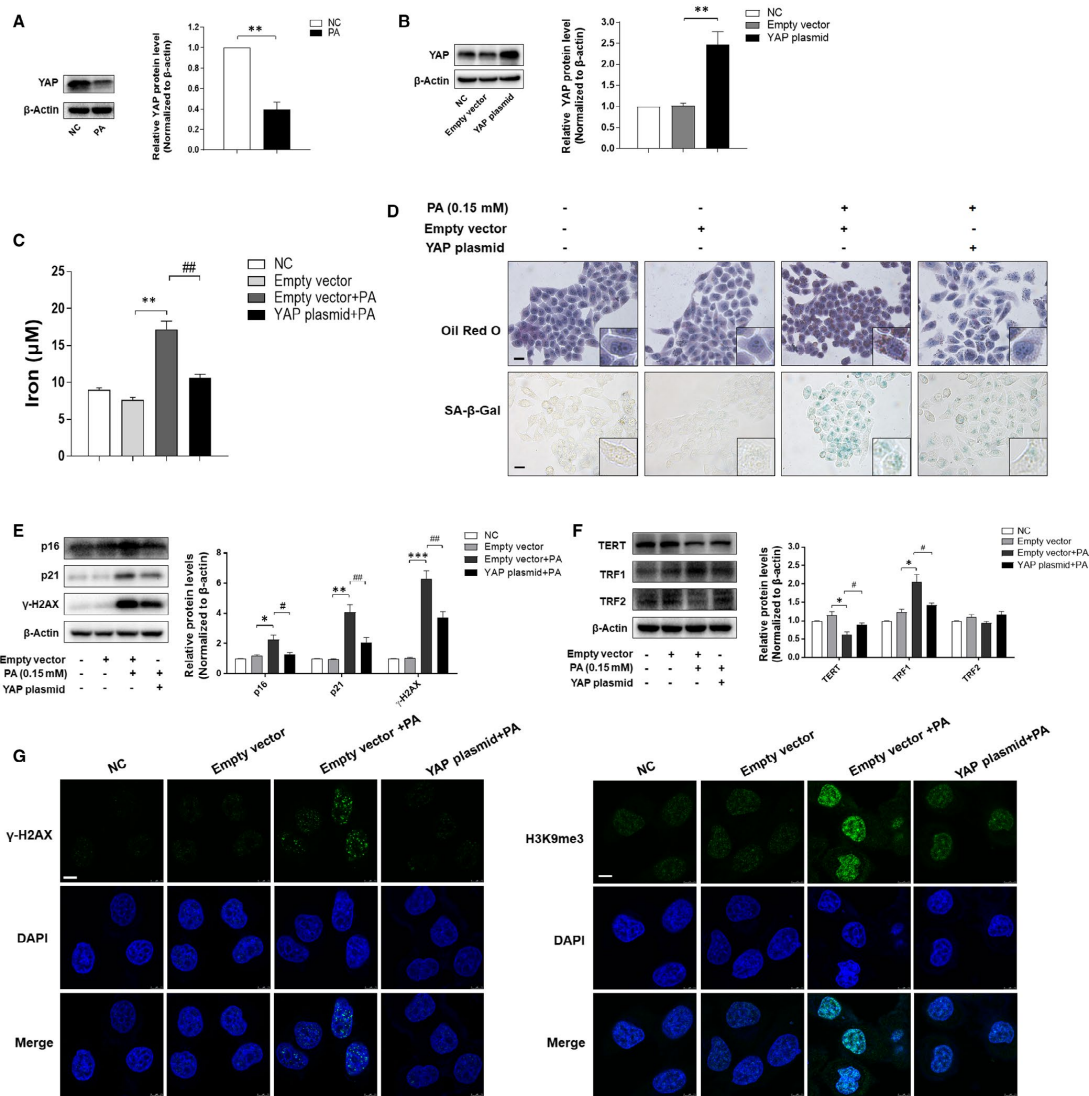
Overexpression of YAP inhibited iron overload and cellular senescence in PA‐treated LO2 cells. A, Western blot analysis of YAP expression in LO2 cells treated with or without PA. Data are represented as mean ±SEM. Significance: ^∗∗^ *P <*.01 versus NC. B, The transfection efficiency of YAP expression plasmid was measured by Western blot analysis. Data are represented as mean ±SEM. Significance: ^∗∗^ *P <*.01 versus Empty vector plasmid. C, The iron content of LO2 cells was evaluated by Iron Content Assay Kit. Significance: ^∗∗^ *P <*.01 versus Empty vector plasmid, ^##^ *P <*.01 versus Empty vector plasmid +PA. D, Representative image of Oil Red O and SA‐β‐gal staining of LO2 cells under the indicated conditions. Scale bar, 100 μm. E, F, Western blot analyses of protein levels of p16, p21, γ‐H2AX, TERT, TRF1 and TRF2. Data are represented as mean ±SEM. Significance: ^∗^ *P <*.05 versus Empty vector plasmid, ^∗∗^ *P <*.01 versus Empty vector plasmid, ^∗∗∗^ *P <*.001 versus Empty vector plasmid, ^#^ *P <*.05 versus Empty vector plasmid +PA, ^##^ *P <*.01 versus Empty vector plasmid +PA, ^###^ *P <*.001 versus Empty vector plasmid +PA. (G) Immunofluorescence staining analyses of γ‐H2AX and H3K9me3. Scale bar, 7.5 μm

Previous reports indicated that YAP is an important mediator of cellular senescence.^32^, ^33^ We performed a series of experiments to detect whether YAP is related to iron overload and cell ageing in PA‐treated LO2 cells. As illustrated in Figure 4A, the protein level of YAP was reduced under the PA treatment. On the other hand, we transfected LO2 cells with YAP CRISPR Activation Plasmid to up‐regulate the expression of YAP (Figure 4B). As expected, overexpression of YAP could alleviate iron overload in PA‐treated LO2 cells (Figure 4C) and ameliorate PA‐induced intracellular lipid accumulation and cellular senescence (Figure 4D). In accordance with these findings, analyses of Western blot and immunofluorescence staining displayed that overexpression of YAP led to a significant reversion of PA‐induced up‐regulation of senescence markers, including p16, p21, γ‐H2AX and TRF1 (Figure 4E,F). On the other hand, the effect of YAP expression plasmid on TERT from our trial was opposite to the above indicators (Figure 4F). Immunofluorescence staining provided consistent results by detecting the expression of senescence markers of γ‐H2AX and H3K9me3 (Figure 4G). These data suggested that YAP mediated iron overload and cellular senescence in PA‐treated LO2 cells.

### 3.5 | **MAYA drove iron overload and hepatocyte senescence via down‐regulation of YAP protein**

**FIGURE 5:**
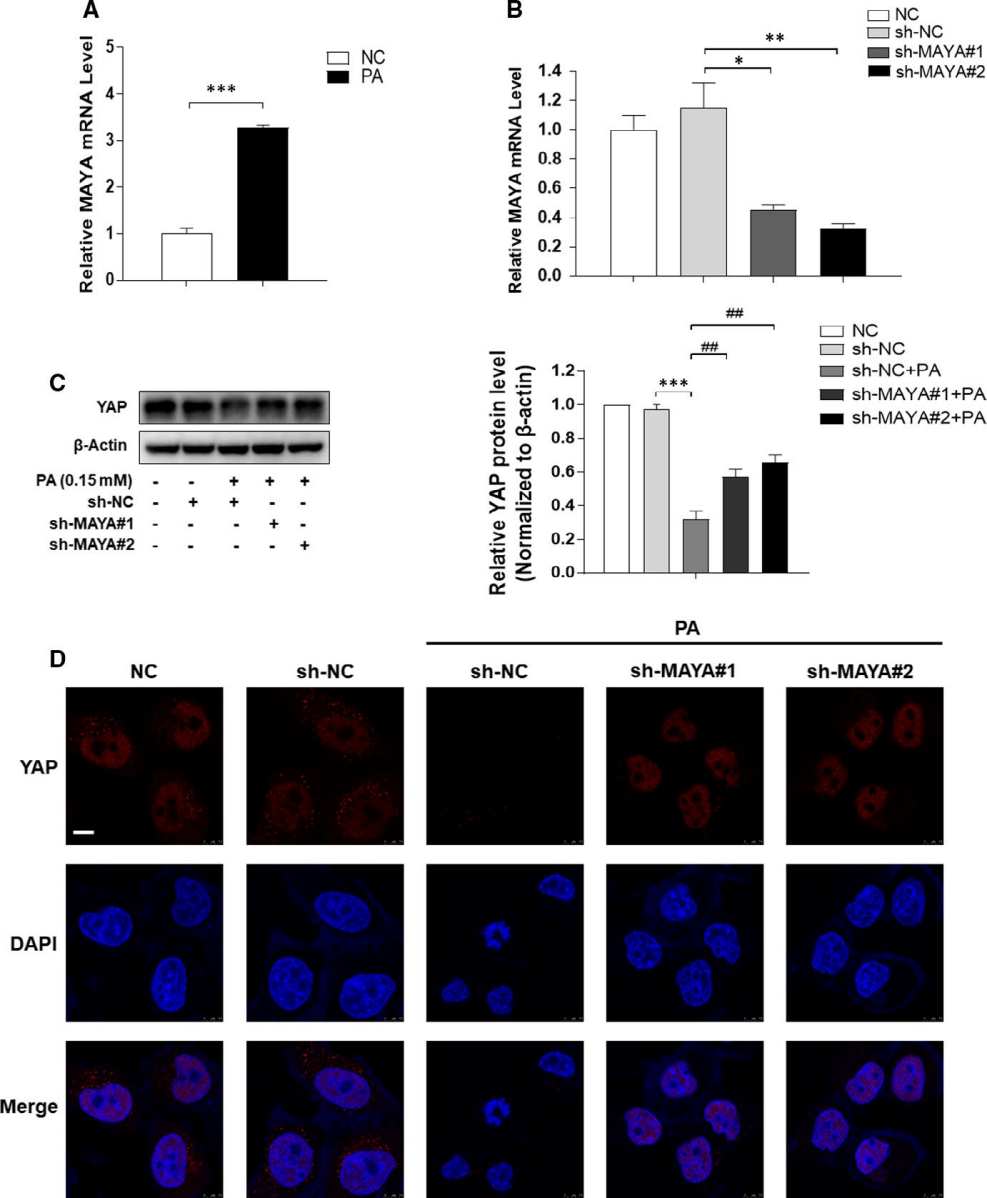
MAYA down‐regulated the expression of YAP in PA‐treated LO2 cells. A, qRT‐PCR analysis the mRNA level of MAYA in LO2 cells treated with or without PA. Data are represented as mean ±SEM. Significance: ^∗∗∗^ *P <*.001 versus NC. LO2 cells were transfected with sh‐NC or sh‐MAYA for 6 h and further incubated with PA (0.15 mmol/L, 24 h). B, MAYA interference efficiency was detected by qRT‐PCR in LO2 cells. ^∗^ *P <*.05 versus sh‐NC, ^∗∗^ *P <*.01 versus sh‐NC. C, D, Western blot and immunofluorescence staining analyses of the expression of YAP. Scale bar, 7.5 μm. Data are represented as mean ±SEM. Significance: ^∗∗∗^ *P* <.001 versus sh‐NC, ^##^ *P <*.01 versus sh‐NC +PA

**FIGURE 6:**
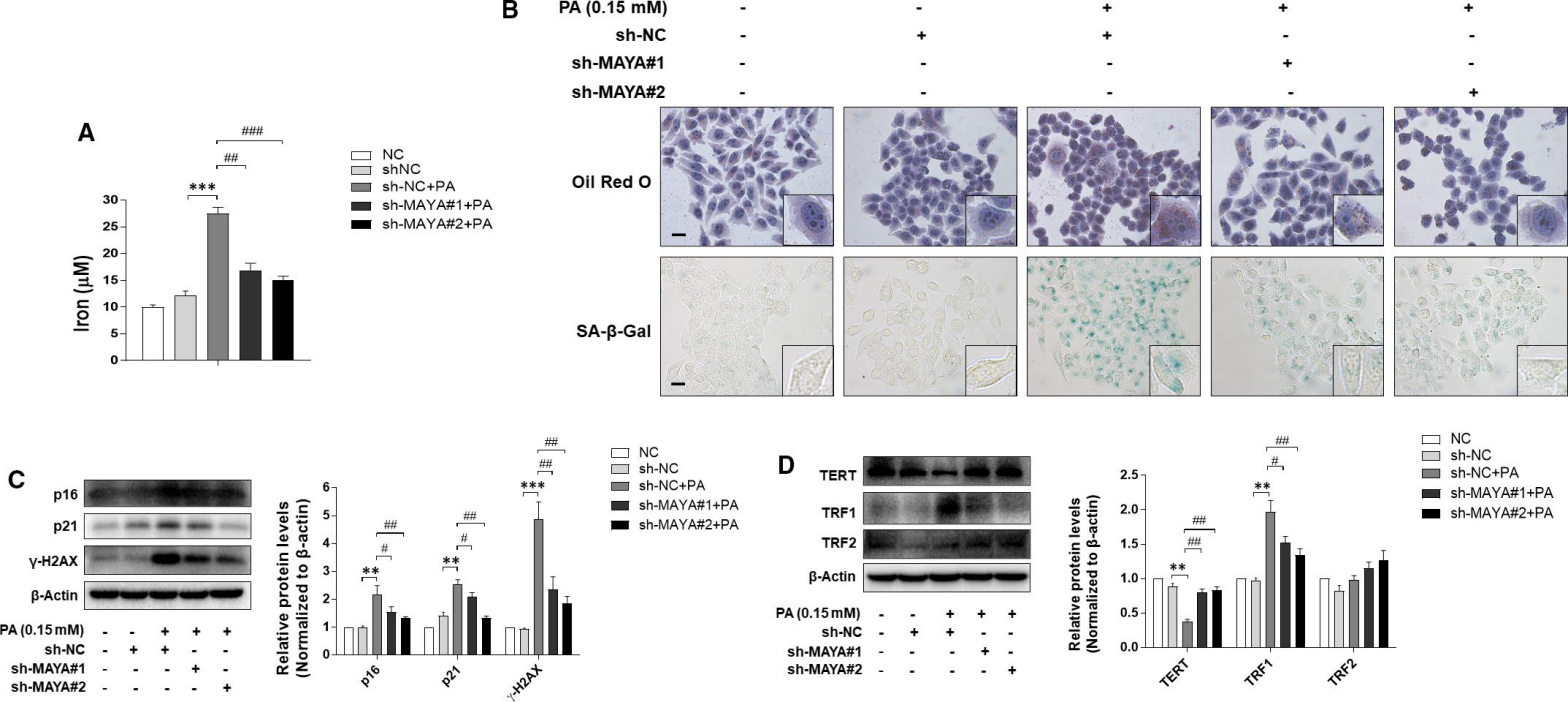
Inhibition of MAYA alleviated PA‐induced iron overload and hepatocyte senescence. LO2 cells were transfected with sh‐NC or sh‐MAYA for 6 h and further incubated with PA (0.15 mmol/L, 24 h). A, The iron content of LO2 cells was evaluated by Iron Content Assay Kit. Significance: ^∗∗∗^ *P <*.001 versus sh‐NC, ^##^ *P <*.01 versus sh‐NC +PA. ^###^ *P <*.001 versus sh‐NC +PA. B, Representative image of Oil Red O and SA‐β‐gal staining of LO2 cells under the indicated conditions. Scale bar, 100 μm. C, D, Western blot analyses of protein levels of p16, p21, γ‐H2AX, TERT, TRF1 and TRF2. Data are represented as mean ±SEM. Significance: ^∗∗^ *P* <.01 versus sh‐NC, ^∗∗∗^ *P* <.001 versus sh‐NC, ^#^ *P <*.05 versus sh‐NC +PA, ^##^ *P <*.01 versus sh‐NC +PA

LncRNAs are involved in regulating RNA, DNA and protein interactions and further modulates intracellular signalling pathways.^34^ LncRNA MAYA (MAYA), also known as MNX1‐AS1 (LOC645249), (binds MST1, LLGL2, SAV1, MOB1, NSUN6 and RASSF1) has been reported to mediate the Hippo/YAP pathway by methylating Hippo/MST1 at Lys59.^19^ Our previous data showed that the mRNA level of MAYA was enhanced in PA‐treated LO2 cells (Figure 5A). We here assumed that inhibition of MAYA might stimulate the expression of YAP, which in turn reduced hepatocyte senescence and ultimately improved NAFLD. To confirm this assumption, we down‐regulated the level of MAYA by shRNA silencing, and the results indicated that transfection with sh‐MAYA#1 and sh‐MAYA#2 successfully rescued the deficiency of YAP in PA‐treated LO2 cells (Figure 5B, C, D). Consistently, suppression of MAYA expression resulted in a marked amelioration of iron overload, intracellular lipids deposition and cellular senescence in PA‐treated LO2 cells (Figure 6A, 6B), with the levels of senescence marker p16, p21 and γ‐H2AX decreased evidently (Figure 6C). In addition, inhibition of MAYA reduced the protein level of TRF1, whereas the protein level of TERT was elevated significantly (Figure 6D). Taken together, these data collectively indicated that knock‐down of MAYA alleviated PA‐induced iron overload and hepatocyte senescence via up‐regulation of YAP protein.

### 3.6 | **MAYA promoted HFD‐induced iron overload and hepatocyte senescence via inhibition of YAP in vivo**

**FIGURE 7:**
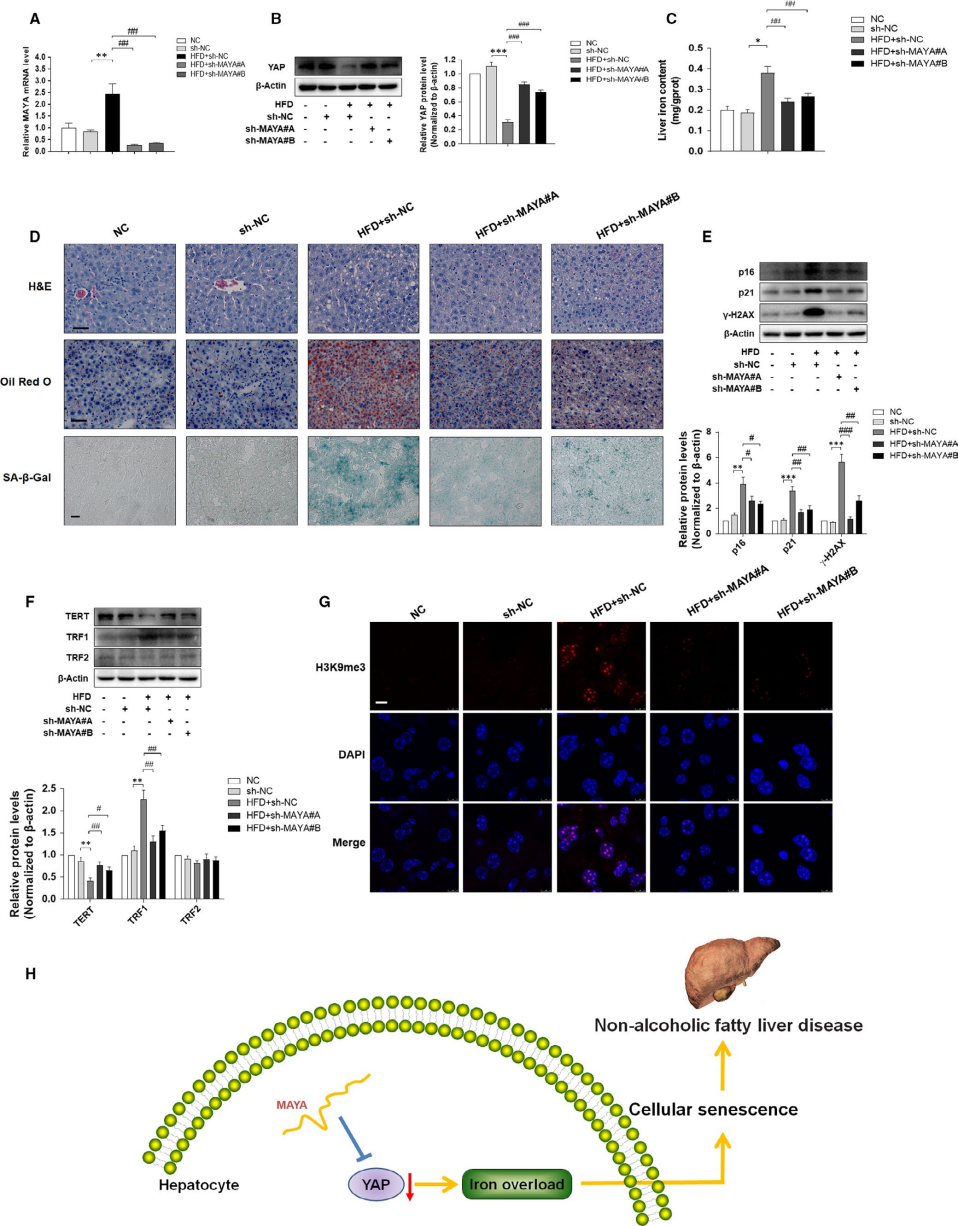
MAYA promoted HFD‐induced iron overload and hepatocyte senescence via inhibition of YAP in vivo. C57BL/6J mice were grouped into the following five categories: group 1, NC group (a standard diet); group 2, sh‐NC group (a standard diet, Lentivirus‐mediated sh‐NC); group 3, HFD +sh‐NC group (a high‐fat diet, Lentivirus‐mediated sh‐NC); group 4, HFD +sh‐MAYA#A group (a high‐fat diet, Lentivirus‐mediated sh‐MAYA#A); group 5, HFD +sh‐MAYA#B group (a high‐fat diet, Lentivirus‐mediated sh‐MAYA#B). A, qRT‐PCR analysis of MAYA mRNA level in the mice' liver. Data are represented as mean ±SEM. Significance: ^∗∗^ *P <*.01 versus sh‐NC, ^##^ *P <*.01 versus HFD +sh‐NC. B, Western blot analysis of the protein expression of YAP in the mice' liver. Data are represented as mean ±SEM. Significance: ^∗∗*^ *P <*.001 versus sh‐NC, ^###^ *P <*.001 versus HFD +sh‐NC. C, The iron content of liver was evaluated by Tissue Iron Colorimetric Assay Kit. Significance: ^∗^ *P <*.05 versus sh‐NC, ^##^ *P <*.01 versus HFD +sh‐NC. D, Liver sections were stained with H&E, Oil Red O and SA‐β‐gal. Representative pictures are shown. Scale bar, 50 μm (H&E and Oil Red O), 100 μm (SA‐β‐gal). E, F, Western blot analyses of the protein levels of p16, p21, γ‐H2AX, TERT, TRF1 and TRF2. Data are represented as mean ±SEM. Significance: ^∗∗^ *P <*.01 versus sh‐NC, ^∗∗∗^ *P <*.001 versus sh‐NC, ^#^ *P <*.05 versus HFD +sh‐NC, ^##^ *P <*.01 versus HFD +sh‐NC, ^###^ *P <*.001 versus HFD +sh‐NC. G, Immunofluorescence staining analysis of the expression of H3K9me3. Scale bar, 7.5 μm. H, Suppression of MAYA had the capacity to reduce cellular senescence and ameliorate NAFLD, which might be related to the regulation of YAP and subsequent alleviation of iron overload

Finally, to determine the exact role of MAYA in regulating iron overload and cellular senescence under HFD condition, C57BL/6J mice were transfected with lentivirus‐mediated sh‐MAYA, followed by normal/HFD treatment. As shown in Figure 7A, MAYA was successfully suppressed by sh‐MAYA#A and sh‐MAYA#B in the liver of HFD‐fed mice. Further assays showed that MAYA silencing reversed HFD‐induced suppression of YAP in mice (Figure 7B), accompanied by a significant amelioration of iron overload, hepatic steatosis, lipid accumulation and hepatocyte senescence via iron content assay, H&E, Oil Red O staining and SA‐β‐gal staining (Figure 7C,7D). In line with the above mentioned results, inhibition of MAYA led to a similar regulation of senescence‐ and telomere‐ related factors as in in vitro experiments (Figure 7E, F). In addition, the overexpression of heterochromatin marker H3K9me3 was also significantly decreased by sh‐MAYA#A and sh‐MAYA#B treatment in the liver of HFD‐fed mice (Figure 7G). Overall, these data revealed that MAYA promoted HFD‐induced iron overload and hepatocyte senescence via inhibition of YAP in NAFLD mice.

## 4 DISCUSSION

NAFLD, characterized by excessive accumulation of hepatic lipids, is closely linked to non‐alcoholic steatohepatitis (NASH), cirrhosis and hepatocellular carcinoma.^35^ Although the pathogenesis of NAFLD have been extensively studied, the mechanisms associated with cellular senescence are not characterized.^36^ Therefore, understanding the molecular mechanisms of cellular senescence will help find ways to improve NAFLD. At the beginning of this study, we verified whether there exists hepatocyte senescence in NAFLD by in vivo and in vitro experiments. We chose golden hamster as the in vivo model of NAFLD because its lipid distribution and metabolism are very similar to humans. In addition, golden hamsters are prone to form NAFLD model because they are sensitive to cholesterol. The phenotype of senescent cells is characterized by expression of SA‐β‐gal (a lysosomal enzyme), a terminal cell cycle arrest, progressive telomere shortening and activation of a DNA damage response.^37^, ^38^ The cell cycle arrest of senescent cells is mainly manifested by increased expression of cell cycle inhibitors, such as p16 and p21.^4^ γ‐H2AX is regarded as a marker of early DNA damage, which is a characteristic of cellular senescence.^39^, ^40^ Besides, the formation of H3K9me3‐containing heterochromatin foci is associated with the progression of cellular senescence.^41^ Prior studies have shown that mutations in members of the telomerase complex and accumulating of DNA damage exist in NAFLD.^41^, ^42^ We herein confirmed that hepatocyte senescence in in vivo and in vitro NAFLD models by detecting the above series of ageing indicators. Moreover, these results would help us further clarify its potential mechanism.

Iron, an effective pro‐oxidant, participates in the transport of oxygen and the process of tissue respiration and maintains the body's homeostasis. Iron overload could induce an imbalance of the homoeostatic mechanisms, which contributes to liver damage resulting from glucose and lipid metabolism disorders.^43^ In addition, iron overload damages cells and causes cellular senescence through oxidative stress.^13^ However, it is not clear whether iron overload can regulate hepatocyte senescence in NAFLD. In this report, we confirmed that iron deposition existed in the liver of HFD‐treated hamsters and PA‐treated LO2 cells. Then, we used iron overload agonist FAC to clarify whether the regulation of iron overload can reduce the senescence of hepatocyte in NAFLD. We found that FAC enhanced iron deposition and lipid accumulation in PA‐treated LO2 cells. Moreover, senescence marker (SA‐β‐Gal activity, p16, p21), DNA damage marker (γ‐H2AX), the telomere length and telomerase activity (TRF1, TERT) were significantly different from only PA‐ or FAC‐treated LO2 cells, suggesting that PA‐induced cellular senescence was aggravated by iron overload. Therefore, the inhibition of iron overload could be an effective target for the reduction in hepatocyte senescence in NAFLD. Certainly, additional study will be needed to illustrate the detailed mechanism of iron overload regulating hepatocyte senescence.

YAP, the downstream effector of Hippo pathway, played an important role in regulation of cellular senescence. In this work, PA treatment could reverse the high level of YAP in normal LO2 cells. Therefore, a question was raised about whether YAP could reduce cellular senescence by regulation of iron overload in PA‐treated LO2 cells. We herein found that the effect of PA on iron deposition was weakened by YAP expression plasmid. More strikingly, overexpression of YAP could subsequently function as a negative feedback regulator of lipid accumulation. Furthermore, restoration of YAP expression attenuated the pro‐senescence effects of PA in cultured LO2 cells. Taken together, these data revealed that up‐regulation of YAP causally inhibited iron overload, which critically led to the suppression of cellular senescence in PA‐treated LO2 cells.

Recently, MAYA have been shown to regulate the activation of YAP indirectly in tumour cells.^19^, ^44^ There is another question of whether MAYA regulates the expression of YAP in in vitro model of NAFLD. In this study, our evidence showed that PA treatment increased the mRNA level of MAYA compared with untreated LO2 cells. Subsequently, we used MAYA shRNA to examine the effect of MAYA on the expression of YAP in PA‐treated LO2 cells. The results showed that low level of YAP in sh‐NC with PA‐treated cells was elevated by knockdown of MAYA. Moreover, our data suggested that the effects of PA on iron overload, lipid accumulation and cellular senescence could be reversed by the inhibition of MAYA in LO2 cells. To provide further evidence for a functional role of MAYA in regulation of hepatocyte senescence in in vivo model of NAFLD, lentivirus‐mediated shRNAs targeting the mice MAYA were administered to the mice via tail vein. We found that the effect of HFD on YAP expression was abrogated by silencing MAYA. Lentivirus‐mediated suppression of MAYA alleviated the HFD‐induced hepatic steatosis and cellular senescence in NAFLD mice. These results were consistent with the experimental data in vitro. However, the molecular mechanism on MAYA regulation of YAP has not been comprehensively studied.

In conclusion, the aggregate data in this study indicated that suppression of MAYA had the capacity to reduce cellular senescence and ameliorate NAFLD, which might be related to the regulation of YAP and subsequent amelioration of iron overload (Figure 7H). However, these results do not rule out the possible involvement of any other signalling mechanisms in the hepatocyte senescence of NAFLD. Our findings suggested a novel lncRNA‐mediated approach to improve NAFLD through modulation of hepatocyte senescence.

## CONFLICT OF INTEREST

The authors declare that they have no conflict of interest.

## AUTHOR CONTRIBUTION

**Ping Yuan:** Investigation (lead). **Xiaoyu Qi:** Formal analysis (equal). **Anping Song:** Methodology (equal). **mingyue Ma:** Methodology (equal). **Xinbei Zhang:** Investigation (equal). **Chunfeng Lu:** Formal analysis (equal). **Mianli Bian:** Formal analysis (equal). **Naqi Lian:** Writing‐review & editing (equal). **Jianlin He:** Writing‐review & editing (equal). **Shuguo Zheng:** Conceptualization (equal). **Huanhuan Jin:** Conceptualization (equal).

## Data Availability

The data used to support the findings of this study are available from the corresponding author upon request.
